# Spatial and temporal intracerebral hemorrhage patterns in Dutch-type hereditary cerebral amyloid angiopathy

**DOI:** 10.1177/17474930211057022

**Published:** 2021-11-18

**Authors:** Sabine Voigt, Siham Amlal, Emma A Koemans, Ingeborg Rasing, Ellis S van Etten, Erik W van Zwet, Mark A van Buchem, Gisela M Terwindt, Marianne AA van Walderveen, Marieke JH Wermer

**Affiliations:** 1 Department of Neurology, Leiden University Medical Center, the Netherlands; 2 Department of Biomedical Data Sciences, Leiden University Medical Center, the Netherlands; 3 Department of Radiology, Leiden University Medical Center, the Netherlands

**Keywords:** Cerebral amyloid angiopathy, cerebral hemorrhage, Dutch-type CAA, epidemiology, hemorrhage, intracerebral hemorrhage

## Abstract

**Aim:**

To investigate whether there is a topographical and temporal pattern of index and recurrent intracerebral hemorrhages (ICH) in Dutch-type hereditary Cerebral Amyloid Angiopathy (D-CAA) to increase our understanding on CAA-related ICH development.

**Methods:**

We included patients with DNA confirmed D-CAA or a history with ≥1 lobar ICH and ≥1 first-degree relative with D-CAA. Topographical pattern was studied by location (proportion frontal/parietal/temporal/occipital; infra/supratentorial and occurrence ratios relative to lobe volume) and volume of index and recurrent ICHs were determined on CT. Temporal pattern was examined by time between recurrent ICHs was retrieved from medical records.

**Results:**

We included 72 patients with D-CAA (mean age at index ICH 55 years) with in total 214 ICH. The median follow-up time was 7 years (range 0.8 to 28 years). All ICH were lobar and supratentorial. The index ICH was most frequently located in the occipital lobe (34% vs. 22% in the other three lobes; with index ICH occurrence ratios relative to lobe volume of 1.9 for occipital, 1.0 for temporal, 1.2 for parietal, and 0.5 for frontal, p = 0.001). In 16/47 (34%) patients with multiple ICH, the second ICH was located in the same lobe as the index ICH. The median time-interval between subsequent ICH was #1-2 ICH 27 months, #2-3 ICH 14 months, and #3-4 ICH 7 months (p = 0.6) There was no difference in volume between index and recurrent ICHs.

**Conclusions:**

We found that index and recurrent ICHs in D-CAA have a preference for the occipital lobe and are least frequent in the frontal lobe, which adds to the existing knowledge of histopathological studies on amyloid load in CAA. Surprisingly, there was no acceleration in time nor gradual increase of hematoma volume between subsequent ICHs.

## Introduction

Sporadic cerebral amyloid angiopathy (sCAA) accounts for approximately one third of all primary intracerebral hemorrhages (ICH) worldwide.^1^ Dutch-type hereditary CAA (D-CAA) is an autosomal dominant disease caused by a mutation in the amyloid-β precursor gene.^2^ In D-CAA, the amyloid angiopathy is pathologically, biochemically, and radiologically similar to sCAA.^3^ Most patients with D-CAA develop their first ICH around the age of 55 years when the effect of age-related vascular risk factors is still relatively limited. D-CAA can be confirmed genetically which enables research into CAA development in living patients with a definite diagnosis.

A previous study on hemorrhagic clustering in sCAA found that hemorrhagic lesions occur preferentially in the temporal and/or occipital lobes.^4^ However, in this study none of the patients had a definite diagnosis of CAA and no differentiation was made between microbleeds and ICH, even though these are distinct entities.^5^

sCAA has the highest recurrence risk of all stroke subtypes.^6^ A recent study showed that the incidence rate of recurrent ICH was 20.9 for D-CAA and 8.9 for sporadic CAA per 100 person-years.^7^ However, it is unknown whether these recurrences have a preference for the same lobe as the index ICH and whether there is a pattern in the time course or severity of the event between recurrences.

As CAA has the highest recurrence rate of all stroke subtypes, it is important to inform patients on whether there is an indication for acceleration in time or severity of recurrent events. We hypothesize that ICHs in D-CAA are more often located in the temporal and occipital lobes comparable to sCAA and that acceleration in time occurs because of disease progression.

We investigated spatial and temporal patterns of index and recurrent ICH in D-CAA to increase our understanding on CAA-related ICH development.

## Methods

The dataset analyzed in this study is not publicly available because of restricted access. Further information about the dataset is available from the corresponding author on reasonable request.

We retrospectively included patients with D-CAA from a database which includes all consecutive persons who visited the (outpatient) clinic of the Leiden University Medical Center (LUMC), the national referral center for D-CAA. Patients were diagnosed by DNA analysis or when they had a history of ≥1 lobar ICH and ≥1 first-degree relative with D-CAA. Patients were excluded when no imaging was available. Patients who had multiple simultaneous index ICH in >1 lobe were excluded, since we were not able to define the order in which the bleedings had occurred. The study was approved by the Medical Ethical Committee of the LUMC, who concluded that it did not fall under the medical research on human aspects act.

Location of ICH was classified as frontal, parietal, occipital, or temporal. The borders of the cerebral lobes were defined according to previously published neuroimaging criteria.^8-10^ In case ICHs involved more than one lobe, the location was assigned to the lobe covering the highest volume. Two independent observers (SV and SA) assessed the ICH location. Non-concordant findings were discussed with a third observer with >15 years of experience in the field (MAAvW) to obtain consensus. To compare the spatial pattern of ICH in D-CAA to non-hereditary CAA, we also assessed ICH location in a group of 80 sporadic CAA patients, diagnosed with probable CAA according to the Boston Criteria.^11^

Volume was assessed with the ABC/2 prediction formula.^12^ In patients who had ≥1 recurrent ICH, volume differences between the first ICH and recurrent ICH were classified as: larger or smaller (>1cm3 difference) or comparable (≤1 cm^3^ difference) than the previous ICH.

### Statistical analysis

We used descriptive statistics for the distribution and volumes of ICHs. Since all lobes have different volumes and therefore a different a priori chance for ICH occurrence, a multinomial Chi-Square Goodness-of-Fit analysis was used to assess whether frequencies of index and recurrent ICHs within the four lobes differed from what would be expected by chance if hemorrhages were distributed throughout the cerebrum according to the relative cortical volumes.^13^ We drew a survival plot to assess time until the next ICH, dependent on number of previous ICH and performed a robust log rank test. A Fisher’s exact test of independence was performed to assess a potential association between location of index and recurrent ICHs.

## Results

We included 72 patients with D-CAA with a total number of 214 ICHs (Table 1). Total follow-up time was 561 years (mean FU per patient was 8 years, range 0.8–26 years). All ICHs were lobar and supratentorial. ICHs were most frequently located in the occipital and frontal lobe (Table 1). However, calculated according to relative cortical volume, the occipital lobe was most frequently affected with ratios relative to lobe volume of 1.5 for occipital, 1.2 for temporal, 0.9 for parietal, and 0.7 for frontal (p < 0.0001). The index ICH was located most frequently in the occipital lobe, persisting after correction for relative cortical volume (1.9 p = 0.001) (Table 2). In 16/47 (34%) patients with ≥2 ICH, the first recurrent ICH was located in the same lobe as the index ICH. The interobserver variation (Kappa statistic) for ICH location was almost perfect (0.98).

**Table 1.** Characteristics of the participants

	D-CAA (n = 72)	sCAA (n = 80)
Demographics		
Women	35 (49%)	34 (43%)
Mean age at index ICH (range, SD)	55 (39–78, 8.0)	70 (55–86, 6.5)
DNA proven D-CAA	52 (72%)	0 (0%)
Median number of ICH (range)	2 (1–8)	1 (1–5)
Case-fatality CAA-related ICH	17 (24%)	7 (9%)
Number of ICH		
1	72 (100%)	80 (100%)
2	49 (68%)	26 (33%)
3	30 (42%)	8 (10%)
4	19 (26%)	2 (3%)
5	8 (11%)	1 (1%)
6	4 (6%)	0 (0%)
7	3 (4%)	0 (0%)
8	2 (3%)	0 (0%)
Vascular risk factors		
Hypertension^a^	28 (39%)	52 (65%)
Hypercholesterolemia^b^	32 (44%)	43 (54%)
Diabetes Mellitus type 2	12 (16%)	7 (9%)
Smoking (current and/or past)	37 (51%)	46 (58%)
Location of index ICH	n = 68	*n = 80*
Parietal lobe	15 (22%)	20 (25%)
Occipital lobe	23 (34%)	20 (25%)
Temporal lobe	15 (22%)	11 (14%)
Frontal lobe	15 (22%)	28 (35%)
Cerebellum	0 (0%)	1 (1%)
Location of all ICHs	n = 214	*n = 117*
Parietal lobe	36 (17%)	28 (24%)
Occipital lobe	59 (28%)	30 (26%)
Temporal lobe	58 (27%)	20 (17%)
Frontal lobe	61 (28%)	37 (32%)
Cerebellum	0 (0%)	1 (1%)
Deep	0 (0%)	1 (1%)

ICH: intracerebral hemorrhage; D-CAA: Dutch-type hereditary cerebral amyloid angiopathy; sCAA: sporadic cerebral amyloid angiopathy.

a ≥140/90 mmHg and/or use of antihypertensive drugs.

b Reported in medical history and/or use of statin.

**Table 2.** D-CAA: Distribution of intracerebral hemorrhages adjusted for relative cortical volume estimates

	Frontal	Parietal	Temporal	Occipital	p-value
Relative cortical volumes^13^ (%)	41.0	19.0	22.0	18.0	
Observed distribution of all 214 ICHs (%)	28.5	16.8	27.1	27.6	0.001^a,b^
Distribution ratio: observed/expected	0.7	0.9	1.2	1.5	
Observed distribution of 68 index ICHs (%)	22.1	22.1	22.1	27.6	<0.0001^a,b^
Distribution ratio: observed/expected	0.5	1.2	1.0	1.9	

a p-value from Chi-Square Goodness-of-Fit Test.

b Null hypothesis: ICHs are uniformly distributed over four lobes according to relative cortical lobe volumes.

In the sCAA group, ICHs were located most frequently in the frontal lobe (Table 1). However, after correction for relative cortical volume all ICHs were located most frequently in the occipital lobe (ratio 1.3; p = 0.027, Table 3).

**Table 3.** sCAA: Distribution of intracerebral hemorrhages adjusted for relative cortical volume estimates

	Frontal	Parietal	Temporal	Occipital	p-value
Relative cortical volumes^13^ (%)	41.0	19.0	22.0	18.0	
Observed distribution of all 115 ICHs (%)^c^	32.2	24.3	17.4	26.1	0.027^a,b^
Distribution ratio: observed/expected	0.7	1.1	0.7	1.3	
Observed distribution of 79 index ICHs (%)^c^	35.4	25.3	13.9	25.3	0.074^a,b^
Distribution ratio: observed/expected	1.1	1.7	0.8	1.8	

a p-value from Chi-Square Goodness-of-Fit Test.

b Null hypothesis: ICHs are uniformly distributed over four lobes according to relative cortical lobe volumes.

c ICHs that were not located in one of the four lobes were excluded (n = 2).

**Figure 1.**
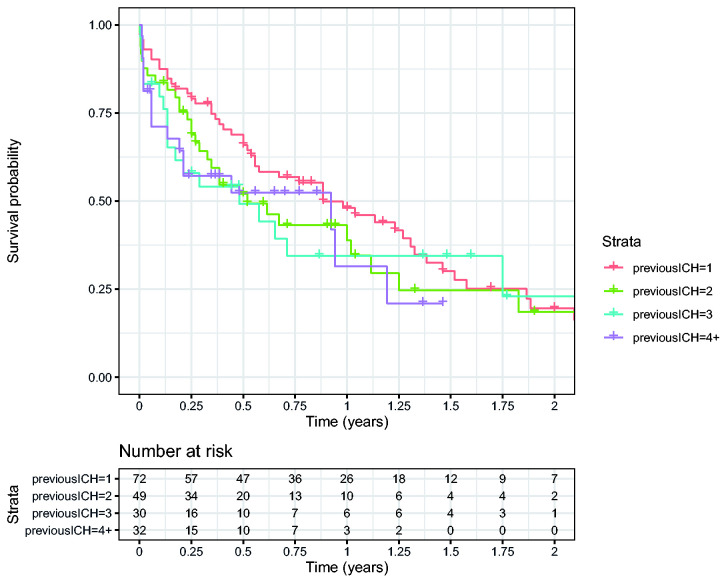
Survival plot showing time until next ICH, dependent on number of previous ICH.

The median time-interval between the ICHs was 27 months between the first and second, 14 months between the second and third, and 7 months between the third and fourth (p = 0.6, Figure 1). Volume assessment was possible in 122 out of 170 ICHs (72%). There was no difference in characteristics between the participants with and without volume assessment. The volumes varied between as well as within patients. There was no clear difference in overall median volume between index and recurrent ICHs; index ICH 8.5 (0.4–76.6), second ICH 6.6 (0.1–79.5), third ICH 15.5 (0.1–69.5), fourth ICH 13.9 (0.6–378.1), and fifth ICH 16.6 (0.6–165.6) (Supplemental Figures 1 and 2). In 42% of patients with a second ICH the volume was larger, in 11% comparable and in 47% smaller compared with the index ICH. In general, clinical outcome worsened after a recurrent event (Supplementary Table 1).

## Discussion

In our study, the occipital lobe was the location of preference for ICH in patients with D-CAA. There was no clear acceleration in time between subsequent ICHs, nor were recurrences more severe in terms of hematoma volume.

The preference for the occipital lobe is consistent with histopathological studies in CAA that found the highest amyloid burden in this region.^14,15^ Our results are also in line with a previous study on hemorrhagic clustering in sCAA, which found a similar preference for the temporal and occipital lobes.^4^ An explanation for the occipital predominance could be that occipital vessels are anatomically thicker and larger with higher levels of collagen-IV in the basement membrane compared with vessels in other lobes.^16^ Binding proteins such as collagen-IV are able to take up amyloid-β from the interstitial fluid in perivascular channels and promote amyloid accumulation.^17^ Furthermore, cerebral arteries in the posterior circulation have more concentric intimal thickening and less elastin than arteries in the anterior circulation. These vessel wall properties possibly lead to different flow dynamics in the posterior area.^17^

We expected acceleration of hemorrhages over time because we assumed that vessels would get more vulnerable as CAA progresses and amyloid accumulation increases. Although the median time between recurrences decreased, survival analyses showed no clear difference in time intervals. However, formal statistical testing was limited by the relative small numbers of patients. Hematoma volume seemed to be randomly distributed without an increase over time.

Our study has limitations. First, large bleedings were attributed to the lobe that covered the greatest area, which is not per definition the lobe in which the initial bleeding started. Also, because patients with large hemorrhages are more likely to die from their ICH this may have affected the chance that a recurrent ICH was larger than the previous. Second, we did not take loss of volume as result of recurrent hemorrhages into account. Third, we used the ABC/2 prediction formula to calculate ICH volume. Although this method takes hemorrhage shape into account, it might be less accurate in case of irregular hemorrhages with fingerlike projections which are often found in CAA. Fourth, we were not able to take possible hemorrhage growth into account, since we did not have full data on time of symptom onset. However, in our clinical experience most D-CAA patients present to the hospital relatively late and hemorrhage growth often stabilizes within 12 h.^18^ Lastly, we only included symptomatic ICH and did not include asymptomatic macrobleeds. Strong points are our unique genetic cohort with a definite diagnosis and relatively pure form of CAA.

sCAA has the highest recurrence rates of all stroke subtypes. Our results can be used to inform patients that in D-CAA, which is pathophysiologically very similar to sCAA, there is no clear indication for acceleration in time or severity of recurrent events. Further insight in the mechanisms underlying CAA-related ICH is needed to ultimately prevent recurrent bleeding in patients with CAA.

## Supplemental Material

sj-jpg-1-wso-10.1177_17474930211057022 – Supplemental material for Spatial and temporal intracerebral hemorrhage patterns in Dutch-type hereditary cerebral amyloid angiopathy Click here for additional data file. Supplemental material, sj-jpg-1-wso-10.1177_17474930211057022 for Spatial and temporal intracerebral hemorrhage patterns in Dutch-type hereditary cerebral amyloid angiopathy by Sabine Voigt, Siham Amlal, Emma A Koemans, Ingeborg Rasing, Ellis S van Etten, Erik W van Zwet, Mark A van Buchem, Gisela M Terwindt, Marianne AA van Walderveen and Marieke JH Wermer in International Journal of Stroke

sj-jpg-2-wso-10.1177_17474930211057022 – Supplemental material for Spatial and temporal intracerebral hemorrhage patterns in Dutch-type hereditary cerebral amyloid angiopathy Click here for additional data file. Supplemental material, sj-jpg-2-wso-10.1177_17474930211057022 for Spatial and temporal intracerebral hemorrhage patterns in Dutch-type hereditary cerebral amyloid angiopathy by Sabine Voigt, Siham Amlal, Emma A Koemans, Ingeborg Rasing, Ellis S van Etten, Erik W van Zwet, Mark A van Buchem, Gisela M Terwindt, Marianne AA van Walderveen and Marieke JH Wermer in International Journal of Stroke

sj-pdf-3-wso-10.1177_17474930211057022 – Supplemental material for Spatial and temporal intracerebral hemorrhage patterns in Dutch-type hereditary cerebral amyloid angiopathy Click here for additional data file. Supplemental material, sj-pdf-3-wso-10.1177_17474930211057022 for Spatial and temporal intracerebral hemorrhage patterns in Dutch-type hereditary cerebral amyloid angiopathy by Sabine Voigt, Siham Amlal, Emma A Koemans, Ingeborg Rasing, Ellis S van Etten, Erik W van Zwet, Mark A van Buchem, Gisela M Terwindt, Marianne AA van Walderveen and Marieke JH Wermer in International Journal of Stroke
