# Supplementary figures and images for: Spatial and temporal intracerebral hemorrhage patterns in Dutch-type hereditary cerebral amyloid angiopathy
Source: Int J Stroke. 2021 Nov 18;17(7):793–8. doi: 10.1177/17474930211057022 (PMC9373023; doi:10.1177/17474930211057022)

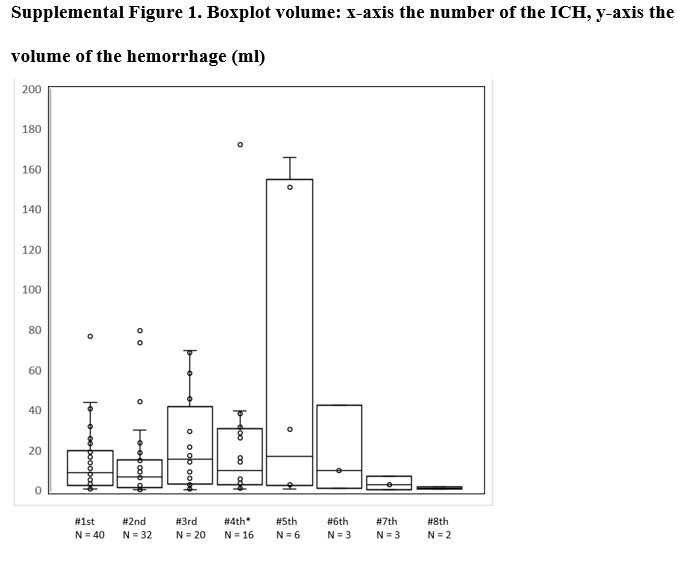

Supplement: sj-jpg-1-wso-10.1177_17474930211057022 – Supplemental material for Spatial and temporal intracerebral hemorrhage patterns in Dutch-type hereditary cerebral amyloid angiopathy [file sj-jpg-1-wso-10.1177_17474930211057022.jpg]

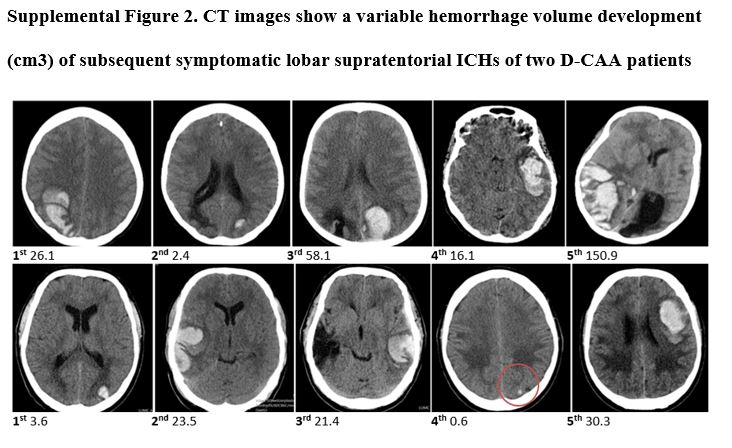

Supplement: sj-jpg-2-wso-10.1177_17474930211057022 – Supplemental material for Spatial and temporal intracerebral hemorrhage patterns in Dutch-type hereditary cerebral amyloid angiopathy [file sj-jpg-2-wso-10.1177_17474930211057022.jpg]
