# Supplementary material for: Spatial and temporal intracerebral hemorrhage patterns in Dutch-type hereditary cerebral amyloid angiopathy
Source: Int J Stroke. 2021 Nov 18;17(7):793–8. doi: 10.1177/17474930211057022 (PMC9373023; doi:10.1177/17474930211057022)
Supplement: sj-pdf-3-wso-10.1177_17474930211057022 – Supplemental material for Spatial and temporal intracerebral hemorrhage patterns in Dutch-type hereditary cerebral amyloid angiopathy [file sj-pdf-3-wso-10.1177_17474930211057022.pdf]

Supplementary Table 1. Discharge destination per event in D-CAA

	#1 ICH (n=72)	#2 ICH (n=49)	#3 ICH (n=30)	#4 ICH (n=19)	#5 ICH (n=8)
Home	31 (43%)	24 (49%)	15 (50%)	5 (26%)	4 (50%)
Rehabilitation clinic	5 (7%)	8 (16%)	4 (13%)	6 (32%)	1 (13%)
Moved to a different hospital	1 (1%)	0 (0%)	1 (3%)	0 (0%)	0 (0%)
Deceased	1 (1%)	2 (4%)	2 (7%)	3 (21%)	2 (25%)
Nursing home	1 (1%)	1 (2%)	2 (7%)	4 (21%)	0 (0%)
No discharge (next ICH occurred during hospitalization)	1 (1%)	1 (2%)	2 (7%)	0 (0%)	0 (0%)
Missing information	33 (46%)	13 (27%)	4 (13%)	1 (5%)	1 (13%)
